# Isolated cerebellar mucormycosis, slowly progressive over 1 year in an immunocompetent patient

**DOI:** 10.4103/2152-7806.73800

**Published:** 2010-12-13

**Authors:** Ellen L. Air, Achala A. Vagal, Ady Kendler, Christopher M. McPherson

**Affiliations:** 1 Department of Neurosurgery, University of Cincinnati College of Medicine, Brain Tumor Center at University of Cincinnati (UC) Cincinnati, OH; 2 Department of Radiology, University of Cincinnati College of Medicine, Brain Tumor Center at University of Cincinnati (UC) Cincinnati, OH; 3 Department of Pathology, University of Cincinnati College of Medicine, Brain Tumor Center at University of Cincinnati (UC), Cincinnati, OH; 4 Department of Neurosurgery, Neuroscience Institute, Cincinnati, OH; 5 Department of Pathology, Mayfield Clinic, Cincinnati, OH

**Keywords:** Fungal, immunocompetent, infection, intravenous drug abuse, mucormycosis, rhinocerebral

## Abstract

**Background:**

Mucormycosis is a rare, aggressive fungal disease with high mortality, typically presenting as rhinosinusitis in immunocompromised patients.

**Case Description:**

A 43-year-old man with a history of intravenous drug use, Hepatitis C, and no evidence of immunocompromise presented with worsening balance problems. He had received intravenous antibiotics 2.5 years earlier for local infection after injecting heroin into a neck vein. Imaging studies revealed a lesion, likely of neoplastic origin. At resection, purulent fluid sampled by neuropathology revealed right-angled, branching hyphae, suggesting mucormycosis. No further resection was performed, no other disease sites were found, and HIV findings were negative. Two weeks postoperatively, he developed renal failure; intravenous antifungal treatment and hemodialysis were discontinued. When kidney function recovered 2 weeks later, he declined additional treatment.

**Conclusion:**

In our immunocompetent patient, both the location of the infection in the posterior fossa and its slowly progressive characteristic were unique variations of this typically aggressive disease.

## INTRODUCTION

Mucormycosis is a rare fungal disease with an aggressive course and a high mortality, typically presenting as rhinosinusitis in immunocompromised patients.[13,18] Diabetes, long-term steroid use, and immunosuppressive treatment are the greatest risk factors for rhinosinusital disease,[1,14,18,36] as well as isolated intracranial disease from hematologic spread.[33] Active intravenous drug abuse (IVDA) typically precedes acute intracranial infection; a few cases have been identified without either associated IVDA or immunosuppression.[20,30,33] Treatment is generally aggressive surgical debridement and long-term intravenous (IV) amphotericin B. Several case reports have documented resolution with IV antifungal treatment alone.[5,18,24,33]

Consistent with its aggressive nature, patients typically present with acute infection, rapidly progressing over several days.[33] In this unique case, our patient had isolated intracranial mucormycosis presenting with findings similar to a slow-growing mass rather than acute infection.

## CASE REPORT

A 43-year-old man developed worsening balance problems during the previous 12 months. He had suffered a left middle cerebral artery ischemic stroke 20 years earlier, secondary to thoracic gun-shot wound and associated vascular injury. Despite the right hemiparesis, expressive aphasia, and seizure disorder that resulted, he lived independently and held various jobs (e.g., lawn-mowing). He had a history of IVDA and was Hepatitis C (HepC) seropositive but reported no IV drugs for 2 years before presentation (i.e., >1 year before initial symptoms) and he was human immunodeficiency virus (HIV) negative. There was no clinical or laboratory evidence of immunocompromise.

Soon after his initial symptoms, magnetic resonance imaging (MRI) imaging performed at another facility documented encephalomalacia from the earlier stroke but no new abnormalities. With progressive unsteadiness, left-arm clumsiness, headaches, and visual disturbances, the patient could no longer work; so, he was referred to our facility for evaluation.

### Physical examination

The patient was alert, appropriate, well nourished, and groomed. With expressive aphasia and moderate dysarthria at baseline, he answered in short phrases rather than complete sentences. With the exception of a central right facial paralysis, cranial nerves II-XII were intact. His right-sided spastic hemiparesis had been stable since his stroke. No dysmetria was noted and Rhomberg sign was negative. He walked with a spastic, unsteady gait that both the patient and his family felt had worsened significantly during the previous year.

### Imaging

**Figure 1:**
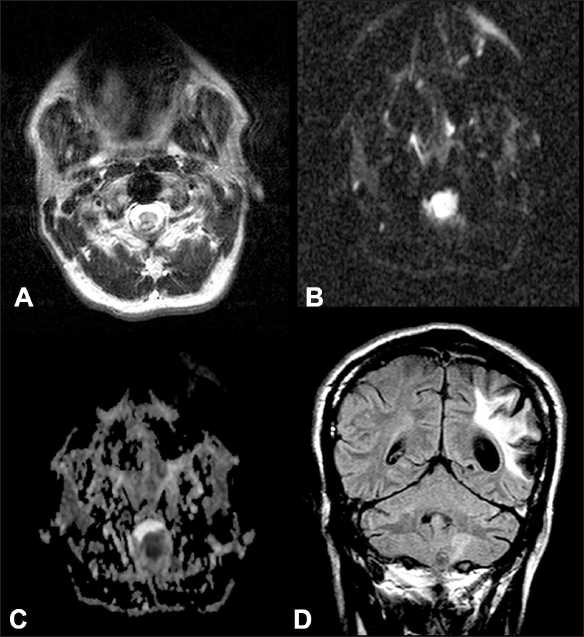
Early MRI evaluation in a 43-year-old man with balance problems and a history of IVDA. T2-weighted axial image at the level of the craniocervical junction (A) with dorsally located hypointensity (arrow) with corresponding true diffusion restriction as seen on diffusion weighted imaging (B), and apparent diffusion coefficient (C) images. Subtle increase in FLAIR signal (D) is noted in the left cerebellar hemisphere (arrowhead). Encephalomalacia from the remote infarct is seen in the left parietal lobe is also seen.

**Figure 2:**
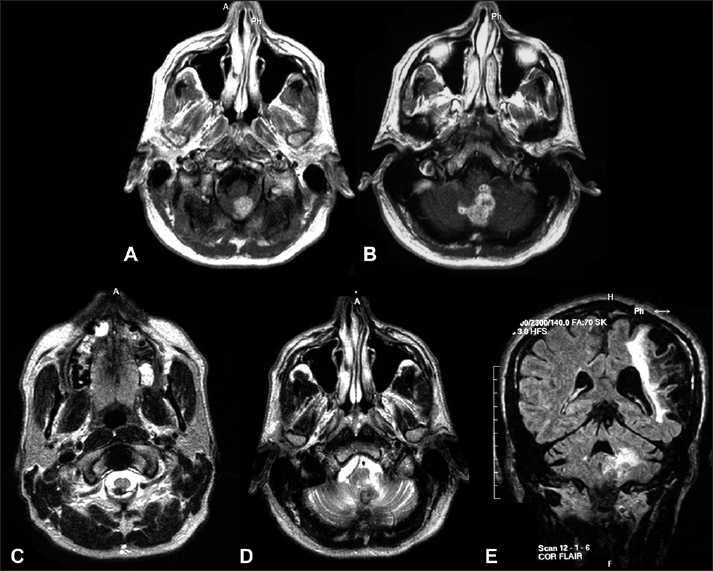
Nine months later, preoperative MRI T1-weighted contrasted axial images showing the enhancing nodular mass in the cerebellar vermis (A) and (B). T2-weighted image showing persistent hypointensity (C) with superior extension (D). Increased FLAIR signal abnormality in the cerebellar hemisphere (E). Before surgery, believed to be neoplastic; at surgery, abnormal tissue with pockets of purulent fluid observed.

After re-review of the initial non-contrast MRI from the first institution 9 months earlier, one author noted a 5 × 7 mm hypointensity at the level of the craniocervical junction dorsal to the spinal cord, with associated diffusion restriction [Figure 1A–C]. On Fluid Attenuated Inversion Recovery (FLAIR) imaging, a subtle edema signal could be appreciated in the cerebellar vermis, without mass effect [Figure 1D]. After another MRI series with and without contrast, a new 3.1 × 2.3 cm irregular-enhancing vermian mass without significant mass effect on the fourth ventricle was noted [Figure 2A and B]. The previously noted T2-weighted hypointensity was unchanged at the craniocervical junction [Figure 2C], whereas additional hypointensity extended superiorly into the cerebellar vermis [Figure 2D]. No diffusion weighted images were obtained as part of this MRI. Cerebellar edema had significantly increased [Figure 2E]. No evidence of hydrocephalus or meningeal disease was observed. Contrast computerized tomography (CT) scans of his chest, abdomen, and pelvis revealed no evidence of systemic disease (not shown).

In reviewing all available images, our multidisciplinary neuro-oncology team agreed that the lesion most likely was neoplastic in origin and recommended resection. The interval between the initial MRI and surgery was 10.5 months, that is, approximately 12 months after symptom onset.

### Operation

Due to the location of the mass, the patient was admitted the night before surgery and a temporary transvenous pacer was placed by cardiology. Electrophysiologic monitoring was established before the patient was secured in a Mayfield head clamp and positioned prone. A suboccipital craniotomy and C1 laminectomy was performed through a midline incision. Immediately on attempting to open the dura in the midline over the cisterna magna, the dura was noted to be very thick and a defined cerebrospinal fluid (CSF) space was not found. Therefore, opening of the dura was approached from over the left cerebellar hemisphere, where initially the cerebellar cortex was easily seen with a clearly defined space between it and the dura. As the dissection moved inferiorly, the dura firmly adhered to the cerebellar cortex. Another approach was made over the right hemisphere with similar result. With careful dissection, the layers could be separated and abnormal tissue was apparent beneath the thickened dura.

During resection of abnormal tissue, which corresponded to the enhancing mass visualized by MRI, a few small pockets of purulent fluid were seen. The fluid was cultured, and a sample of abnormal tissue was reviewed by the neuropathologist (AK). Right-angle, branching hyphae were apparent on a smear prep of the fresh tissue, suggesting the diagnosis of mucormycosis. The remaining abnormal tissue appeared to be scar tissue and was not clearly separable from normal cerebellum. Therefore, further resection could not be safely performed. The dural opening was then closed in a water-tight fashion and the wound closed.

### Postoperative course

**Figure 3:**
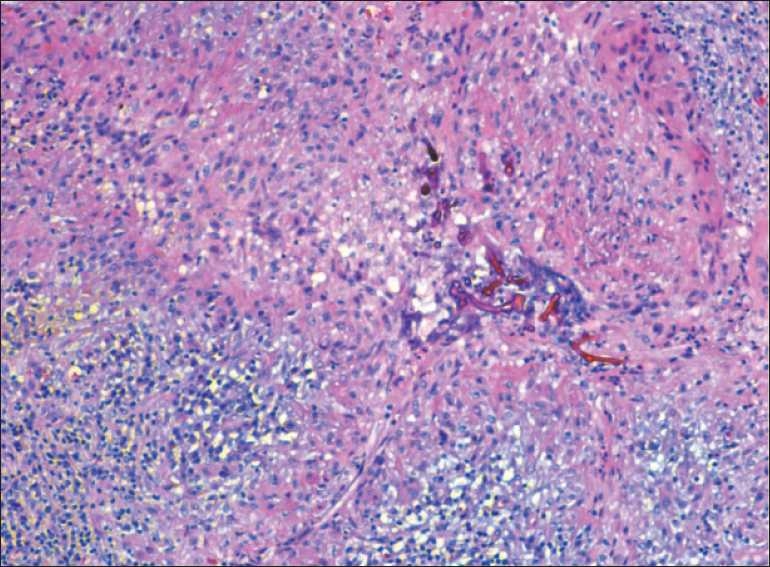
Pathologic sample shows branching hyphae suggesting mucormycosis (arrow). (H&E, ×200)

On pathological findings, broad fungal hyphae were present with random (often 90°) branching and occasional septae [Figure 3]. In addition, dense fibrosis and granulomatous reaction was noted. The patient was observed in the intensive care unit and IV amphotericin B treatment was initiated. In consultation with the Infectious Disease Service, the patient was evaluated for systemic disease or immunosuppression, including an echocardiogram to evaluate for valve vegetations; CTs of the chest, abdomen and pelvis; and MRI of the spine. No other sites of disease were found and HIV findings were negative. Although findings of HepC serology were positive, no evidence of liver failure was noted after liver function tests. No fungi were cultured from the intraoperative sample; however, scarce coagulase-negative staphylococci were present. Vancomycin was added to his regimen.

From inquiries about IVDA, we learned from the patient and his family that he had been hospitalized 2.5 years earlier for a local infection after injecting heroin into a neck vein. The infection resolved with IV antibiotics and no antifungals had been administered. Despite continued IVDA after this infection, he subsequently quit and had not injected drugs for at least 2 years. His family corroborated that he had not otherwise been ill since that hospitalization and had been followed by a primary care physician.

On postoperative Day 3, he was discharged to a skilled nursing facility for long-term IV amphotericin B. Two weeks into therapy, he developed renal failure, leading to cessation of the IV antifungal treatment and hemodialysis. When kidney function recovered 2 weeks later, the patient declined additional treatment and left the nursing facility against medical advice. He returned to the apartment that he shared with his brother, and missed follow-up MRI and appointments. He continues to be ambulatory, but his family reports that his gait is worsening while his health has remained stable.

## DISCUSSION

Based on our review of the literature, this is the first reported case of isolated intracranial mucormycosis that presented in an indolent manner. Mucormycosis is typically documented as an acute, rapidly progressing intracranial infection,[9,11,21,24,26,28,29,33] as well as aggressive in systemic, pulmonary, and rhinosinusital disease.[3,4,12,18] However, chronic forms of pulmonary and rhinosinusitis have also been reported. Madhusudhan *et al*. described four cases of progressive pulmonary mucormycosis that developed over months and were initially diagnosed as bronchogenic carcinoma based on radiographic findings.[15] Chronic mucor rhinosinusitis, with secondary intracranial extension, has been increasingly reported. Rumboldt and Castillo reported a case of prolonged intracranial infection with mucormycosis in a young man who underwent a bone marrow transplant for acute lymphocytic leukemia. Similar to other reports, the patient initially received antibiotics and subsequently amphotericin B after presenting with an acute fever, before a diagnosis of fungal infection was made. When enlargement of the clival and prepontine enhancement was documented by MRI, the patient then received multiple courses of antifungal treatment.[25] Therefore, the indolent behavior of this patient with mucormycosis was most likely related to incomplete treatment rather than the natural course of the disease.

Our patient demonstrated a slow clinical course, showed no clinical signs of infection, and had imaging characteristics that led to our preoperative presumption that the lesion most likely represented a neoplastic process. Retrospectively, we believe that the signs on the preoperative imaging were consistent with fungal infection, specifically T2-weighted hypointensity, owing to the paramagnetic and ferromagnetic elements within the fungi.[8,10,14,23] Diffusion restriction, another characteristic of fungal infection, was also present on the initial MRI. However, no diffusion-weighted images were taken as part of the second MRI. These findings may be characteristic of fungal infections but are not unique and can also be associated with neoplastic lesions.

Our patient’s prolonged course of infection may have been facilitated by his immunocompetent status. Increasing numbers of patients, both immunocompetent and immunocompromised, are afflicted with chronic rhinosinusitis.[17,26] Survival rates after mucormycosis have also increased, up to 60%.[16,19] Survival, which has been reported in several patients who did not undergo surgical debridement,[5,7,13,18] remains largely associated with early diagnosis and isolated rhinocerebral disease,[8] and is not limited to immunocompetent patients. Unfortunately, explaining these trends related to infection and survival rates are difficult, given the relative rarity of mucormycosis and the fact that these fungi do not grow well in culture.[33]

The initial source of infection in our patient remains unclear. In the absence of pulmonary or rhinosinusital disease, a primary fungemia must be assumed. Although our patient had a history of IVDA, a known risk factor for isolated intracranial fungal abscess, he reported that his last use was distant to symptom onset. As for any IVDA case, we questioned the veracity of this time interval and interviewed several sources, including his brother who shared his apartment and ascertained that the patient had no exposures or illnesses for 1 year before symptom onset. The history of neck infection raises the possibility that the mucor was seeded at that time. If the neck infection or later IVDA was the source, then the true duration of indolence is far longer than the year we can document. These factors may also be “red herrings” as intracranial mucormycosis has been documented in the absence of an identifiable pre-disposing factor.[31]

The cerebellar location of infection in our patient is also rare. Others have noted that hematogenously spread, intracranial mucormycosis has a predilection for the basal ganglia.[2,11,22,28,32,33] The concurrence of IVDA and basal ganglia mucormycosis is frequent enough for some to advocate fungal infection as a primary on the list of differential diagnoses in IV drug abusers presenting with basal ganglia lesions.[28] Our case represents one of the very few documented in the posterior fossa. To our knowledge, only one previous case of cerebellar mucormycosis has been reported.[21] Of two patients who have been reported with prepontine lesions, both appeared to be the result of skull base extension.[25,35] Intraventricular and brainstem disease associated with obstructive hydrocephalus have also been documented.[4,34]

Aggressive debridement continues to be the mainstay of mucormycosis treatment. In rhinocerebral cases, this requires exenteration of the sinuses and, possibly, the orbit. Intracranial extension, most often in the frontal lobes, must also be debrided. In our patient, resection was limited owing to significant scarring, the lack of clear tissue planes, and deep extension toward the brainstem. Aggressive debridement in the posterior fossa would have posed a high risk of neurologic decline. The necessity of operative debridement has been debated, whereas the need for aggressive antifungal treatment with IV amphotericin B has not.[8] However, amphotericin B is nephrotoxic even in its liposomal formulation, which our patient received; therefore, treatment must be monitored carefully. After suffering acute renal failure and temporary dialysis, our patient declined further care. Of import, no recommendations yet exist to guide ongoing treatment once a patient has suffered severe nephrotoxicity.

## CONCLUSIONS

Intracerebral mucormycosis remains a challenging and aggressive infection to treat. Once thought to be only an opportunistic infection, intracerebral mucormycosis can take many forms in the immunocompetent patient. In this case report, both the location of the infection in the posterior fossa and the slowly progressive characteristic of this infection in an immunocompetent patient represented a unique variation of this typically aggressive disease.
